# Assessing Boundary Conditions of the Testing Effect: On the Relative Efficacy of Covert vs. Overt Retrieval

**DOI:** 10.3389/fpsyg.2017.01018

**Published:** 2017-06-21

**Authors:** Max L. Sundqvist, Timo Mäntylä, Fredrik U. Jönsson

**Affiliations:** Department of Psychology, Stockholm UniversityStockholm, Sweden

**Keywords:** testing effect, paired-associate learning, cued recall, covert retrieval, overt retrieval

## Abstract

Repeated testing during learning often improves later memory, which is often referred to as the testing effect. To clarify its boundary conditions, we examined whether the testing effect was selectively affected by covert (retrieved but not articulated) or overt (retrieved and articulated) response format. In Experiments 1 and 2, we compared immediate (5 min) and delayed (1 week) cued recall for paired associates following study-only, covert, and overt conditions, including two types of overt articulation (typing and writing). A clear testing effect was observed in both experiments, but with no selective effects of response format. In Experiments 3 and 4, we compared covert and overt retrieval under blocked and random list orders. The effect sizes were small in both experiments, but there was a significant effect of response format, with overt retrieval showing better final recall performance than covert retrieval. There were no significant effects of blocked vs. random list orders with respect to the testing effect produced. Taken together, these findings suggest that, under specific circumstances, overt retrieval may lead to a greater testing effect than that of covert retrieval, but because of small effect sizes, it appears that the testing effect is mainly the result of retrieval processes and that articulation has fairly little to add to its magnitude in a paired-associates learning paradigm.

## Introduction

A wealth of research has shown that individuals who repeatedly test memory during learning will perform better on a later recall test than those who spend an equal amount of time repeatedly studying the same material, a phenomenon often referred to as the testing effect (e.g., Gates, 1917; Carrier and Pashler, 1992; see Roediger and Karpicke, 2006, for a review). This kind of self-testing has several advantages in terms of learning, monitoring and regulation: It acts as a diagnostic test of the ongoing learning process which may in turn help to direct further studying efforts to where they are most needed (Metcalfe, 2009). Perhaps more importantly, it may also boost memory itself, as evidenced by the testing effect.

Although the testing effect itself is a robust phenomenon, its boundary conditions are less well understood. While the testing effect has been found in a multitude of materials (e.g., Wheeler and Roediger, 1992; Carpenter and DeLosh, 2006; Roediger and Karpicke, 2006; Carpenter and Pashler, 2007; Kang et al., 2007; Karpicke and Roediger, 2007), all these findings are based on the same *response format*, namely an overt testing procedure. When tested during learning, participants’ memory is typically assessed by having them overtly articulate the correct answer, for instance by typing it on a keyboard or saying it out loud. If the answer is not articulated, there is no way, experimentally speaking, of scoring these responses. In everyday settings, however, many students will likely engage in retrieval practice that is entirely covert, that is, an answer that is retrieved and produced internally by thinking it, but with no overt articulation of that information. For this reason, it is important to know if there is a relative advantage in terms of the efficacy of these response formats, as it has implications not only for understanding the testing effect itself, but also for the development of optimal learning and teaching instructions. Dunlosky et al. (2013) reviewed the effectiveness of various learning techniques, and found that retrieval practice was among the few that had high utility (i.e., the effect was robust and generalized widely). The testing effect is clearly a robust effect, and it can explain why retrieval practice is of such high utility as a learning technique, but it remains to be seen whether the relative efficacy of covert and overt retrieval is also a robust phenomenon, or if it only exists in experimental settings that do not generalize to real-world settings – if it exists at all. One way of assessing this is to consider effect sizes. If they are small (e.g., Cohen’s *d* < 0.3; Cohen, 1988), the real-world implications are also limited (see **Table 1**).

**Table 1:** The relative efficacy of overt vs. covert retrieval as reported in 13 experiments.

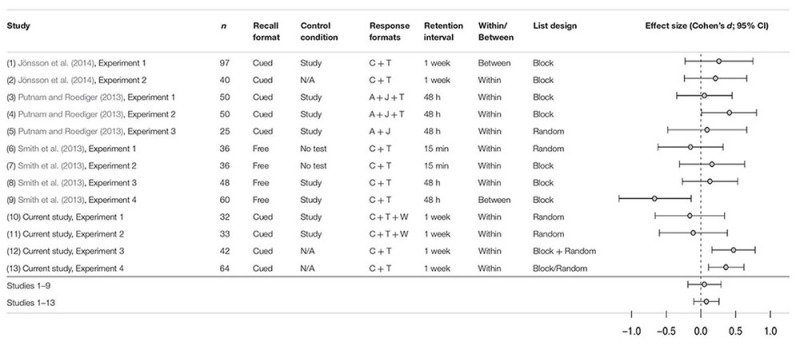

*The response formats used in the experiments include typing (T), saying answers aloud (A), making JOLs (J), handwriting (W), and covert retrieval (C). The within/between distinction refers to whether the response formats were manipulated within or between subjects. List order refers to whether items were tested covertly and overtly in blocks or in random order (note that for between-subjects designs, the list design will always be “block”). A positive effect size indicates that overt retrieval produced a stronger effect than covert retrieval after the specified retention interval. Also note that Experiment 4 in the current study manipulated response format within subject, but varied the list design between subjects. No studies, except Experiments 2 and 3 of Putnam and Roediger (2013) provided feedback during learning. The aggregated effect sizes for Studies 1–9 and 1–13 are were calculated using a random-effects meta-analysis, with each study weighted according to its number of participants. The heterogeneity index for the meta-analysis of all 13 studies was *I*^2^ = 0.56. The Study by Izawa (1976) is not included in this table because it only includes means for each condition, and therefore, its effect sizes cannot be calculated*.

The testing effect has been demonstrated for a large number of materials and testing formats (see Roediger et al., 2010, for a review), although the specific response format of the test (during learning) has received little attention. Most testing-effect experiments utilize either free recall or cued recall in various forms of overt testing. However, some studies have reported a testing effect following a covert retrieval practice (e.g., Carpenter et al., 2006; Carpenter and Pashler, 2007; Carpenter et al., 2008; Kang, 2010; Jönsson et al., 2014).

Similar findings have also been made in metamemory research, where the act of judging the degree to which something has been learned (i.e., judgments of learning, JOLs; Nelson and Dunlosky, 1991) seems to improve memory itself. When a JOL is made after a delay, it elicits an attempted retrieval of the sought-after information, and successful retrieval is associated with a testing effect (Spellman and Bjork, 1992). If the JOL was made immediately after study, the information would likely be available in short-term memory, and therefore no testing effect should be produced because there was no retrieval attempt (e.g., Nelson and Dunlosky, 1991). In other words, delayed JOLs should produce testing effects because they entail covert retrieval (e.g., Sundqvist et al., 2012; Jönsson et al., 2014; Akdoğan et al., 2015; see Rhodes and Tauber, 2011, for a review), although not all studies confirm this. For instance, Tauber et al. (2015) found that while both delayed JOLs and delayed testing entail covert retrieval, delayed JOLs only had a minor effect on final test performance. Most studies on the delayed JOL effect do not directly compare memory performance following covert and overt retrieval, but they nonetheless provide evidence that a testing effect can be produced by covert retrieval alone, which begs the question of whether articulation has something to add to its magnitude.

### Should Response Format Affect the Magnitude of the Testing Effect?

Although the hypothesis of response format as a moderator for the testing effect is relatively novel, many previous studies have examined the relationship between modality and memory (e.g., Penney, 1975). However, these studies are mainly concerned with the modality of presentation, rather than response (Harvey and Beaman, 2007). Gardiner et al. (1977) examined memory performance after having learned words, by either saying them out loud or writing them down, or both, and found that word recognition was more accurate for the participants who had both spoken and written the words, compared to any of the groups that only spoke or wrote the words. Their interpretation of these results was that (successful) retrieval of some information should strengthen the memory trace for that information, and that the various ways of articulating the answer (e.g., by saying it out loud or writing it down) would cause qualitative differences in the recoding of the trace, such that auditory, articulatory, kinesthetic, or visual attributes become part of the trace, depending on the mode of articulation. This line of reasoning is closely related to the *production effect* (e.g., Ozubko and MacLeod, 2010), whereby saying a word aloud during learning can enhance memory, compared to reading it silently. A reasonable explanation of the production effect is that the creation of a verbal cue (that is not present when only reading the word) facilitates future retrieval (MacLeod et al., 2010). Although the production effect is concerned with encoding, rather than retrieval, it is reasonable to suspect that the mechanism driving the production effect could also cause overt retrieval to be more beneficial for memory than covert retrieval. If this is the case, we should also expect this relative advantage for various forms of overt retrieval (which all entail articulation), relative to covert retrieval, in a testing effect paradigm. Like the production effect, the *generation effect* (e.g., Jacoby, 1978; Slamecka and Graf, 1978) also posits enhanced memory performance as a result of articulation. However, as demonstrated by Karpicke and Zaromb (2010), the testing effect and the generation effect differ by mode of retrieval, such that intentional retrieval is more beneficial for retention than generation (or production) under incidental retrieval instructions. That is, it matters whether retrieval is its own goal, or simply some means of completing some other task. Given this account, covert and overt retrieval should produce testing effects of equal magnitudes. So, the production effect and the generation effect, although highly similar, have different implications for the relative efficacy of covert vs. overt retrieval, which is all the more reason to further investigate the testing effects produced by different response formats.

At this point, we may ask why covert and overt retrieval should give rise to testing effects of different magnitudes at all. A possible explanation comes from the *transfer-appropriate processing* (TAP) account of the testing effect (see Roediger and Karpicke, 2006), which states that the degree of congruency, between encoding and retrieval, will increase the likelihood of successful retrieval, and since final testing is virtually always overt, the TAP hypothesis would predict that overt testing should produce a stronger testing effect than covert testing.

Yet another, perhaps less intriguing explanation is simply the amount of time dedicated to processes involved in the retrieval and articulation of information. As mentioned earlier, if the only difference between covert and overt retrieval is the act of articulation, then we may assume that overt retrieval typically should take longer than covert retrieval simply because it takes additional time to articulate the information that has just been retrieved. This time could be regarded as additional exposure to the information itself, which is likely to increase the memory strength for that information, which in turn boosts the testing effect. Naturally, this can be avoided by having equated exposure times for both covert and overt retrieval conditions. Nonetheless, if we were to provide study advice to students on the basis of the findings in the testing effect literature, the explanation for this relative efficacy becomes rather irrelevant; overt testing should be preferred over covert testing, even if the associated benefit is only due to differences in exposure or processing time, simply because what matters is the memorial benefit itself – not the reason why it exists.

There are four studies, of particular relevance to this work, that have investigated the relative efficacy of covert and overt retrieval on the testing effect (Izawa, 1976; Putnam and Roediger, 2013; Smith et al., 2013; Jönsson et al., 2014). Izawa (1976) had subjects undergo cycles of studying and testing, where testing was either silent (covert) or vocalized (overt). At final recall, covert and overt testing conditions performed equally well, meaning that there was no difference in the magnitude of the testing effect produced by covert vs. overt testing (although there were short-term effects of vocalization). Jönsson et al. (2014) found that overt retrieval produced stronger testing effects than covert retrieval, although the effect size was small (Cohen’s *d* = 0.21). Specifically, in the first of two experiments of their study, they found a response format by retention interval interaction, indicating a testing effect. However, the interaction was mainly driven by differences between the study-only and overt response format conditions, and there was no significant difference between covert and overt conditions at the 1-week retention interval. In the second experiment, covert retrieval was compared to overt retrieval in a within-subjects design (as opposed to Experiment 1, which manipulated response format between subjects), and a main effect of response mode was found, such that overt retrieval was more beneficial than covert retrieval in terms of memory performance. Putnam and Roediger (2013) found mixed evidence of response format, such that overt retrieval led to better final recall in only one of three experiments (Experiment 1 of their study failed to replicate a testing effect, as the restudy condition was confounded by the addition of item-wise JOLs following restudy). Smith et al. (2013) found no difference in free recall performance of items that had been tested overtly or covertly during learning. Taken together, these inconclusive results warrant further investigation of the role of response format on the testing effect.

### The Current Study

The purpose of the current study was to explore possible factors that could help explain and reconcile the disparate results within this field (e.g., Putnam and Roediger, 2013; Smith et al., 2013; Jönsson et al., 2014). Given the design and findings of these studies, there were five main considerations that governed the overall design of the four experiments of this paper:

First of all, a covert retrieval condition would need to be included, with which to compare an overt retrieval condition. This comparison was the main focus of this paper, and is therefore included in all four experiments.

Second, a study-only condition would need to be compared to a study-test condition, across a short and a long retention interval, simply to replicate a testing effect. This would serve mainly as a confirmation that the stimulus material and the tests used would indeed produce a testing effect.

Third, there are different overt response format that may affect the outcome differentially, meaning that overt retrieval could be subdivided into two or more conditions, such as typing and handwriting. This was done for two reasons: (i) from point of view of the TAP account of the testing effect, the magnitude of the testing effect may depend of the level of congruency between the circumstances during learning and the circumstances during testing. If response formats differed (or were the same) during learning and final testing, this would allow not only for comparison between covert and overt retrieval, with respect to the testing effect produced, but also within different forms of overt retrieval, or different levels of TAP congruency, and (ii) based on research on the production effect, articulation may be beneficial for memory under certain circumstances, but as evidenced from research on haptics and handwriting, not all forms of articulation may benefit memory the same way (see Mangen and Velay, 2010 for a review). For instance, handwriting appears to be more beneficial to memory than typing on a keyboard (Mangen et al., 2015; although not all studies have found this advantage, e.g., Vaughn et al., 1992) because the level of embodied cognition involved in handwriting is believed to be higher than in the case of typing, and that this makes memory for handwritten information more distinct and rich in terms of sensomotor and visual content. Thus, if the magnitude of the testing effect depends on articulation, through some mechanism that has yet to be fully explicated, we should expect that different modes of articulation will boost the testing effect to different extents, given the findings of Mangen et al. (2015). If articulation does not contribute to the magnitude of the testing effect, we should expect to observe no differences in memory performance between covert and overt retrieval, regardless of how the articulation was carried out in the overt conditions. This was the aim of Experiments 1 and 2.

Fourth, the different response formats needed to be tested either in blocks, as was the case in both Putnam and Roediger (2013), and Jönsson et al. (2014), or in a random order for each trial, as in Experiment 3 of this paper. Rowland et al. (2014) investigated the effects of mixed vs. pure lists in a testing effect paradigm, and found no differences in the magnitude of the testing effects created by either kind of list. However, their finding that the test effect itself is unaffected by list order does not necessarily mean that the relative efficacy of covert vs. overt retrieval is also unaffected by list order. For instance, Jonker et al. (2014) found a production effect for items read either silently or aloud, but only for mixed (i.e., random) and not pure (i.e., blocked) lists. While this finding pertains more to the item-order account (see McDaniel and Bugg, 2008) than the testing effect, it is an example of differences in memory performance as a function of list order. Moreover, the list order manipulation is of interest because it is directly connected to the way participants perceive the tasks of either covertly or overtly retrieving information. In the sense that covert retrieval is identical to overt retrieval – the difference being a lack of articulation – we can reasonably assume that the overt retrieval process, until the point of articulation, is very similar to the covert retrieval process, if not identical. However, built into this assumption is that participants are not able to anticipate whether the information that has just been covertly retrieved will also need to be overtly articulated. In cued-recall tests that present items in blocks of covert or overt tests, participants are very likely to understand that several items will be tested in the same way (i.e., covertly or overtly) until a change takes place, after which the response format will again remain the same for several items. This design creates a possibility for participants to adopt different retrieval strategies, criteria, or thresholds for giving an affirmative response. If covert and overt testing are instead carried out in random order, participants will have no way of knowing whether the information that is initially retrieved will also need to be articulated overtly.

Jönsson et al. (2014) investigated this possibility by comparing response latencies for overt and covert retrieval during learning and found no differences between the two response formats. This would suggest that covert and overt retrieval indeed involve similar retrieval processes, however, two processes that are equal in duration do not necessarily need to be identical in all other regards. Therefore, testing items covertly and overtly either in blocks or in a random order may provide an explanation to the relative efficacy of covert and overt retrieval that does not pertain to the act of articulation. The rationale is that if the testing effect is only driven by retrieval, covert and overt retrieval should create testing effects of equal magnitude, especially in the case of random testing order, for reasons stated above. For tests given in covert and overt blocks, the retrieval process itself might differ by response format. This would explain the advantage for overt retrieval found by Jönsson et al. (2014), as the result of differences in retrieval processes rather than an added memorial benefit by means of articulation. If, on the other hand, this advantage is due to articulation, we should not expect differences between tests given in blocked or random order. This was the aim of Experiment 3.

Fifth, and finally, the distinction between blocked and random testing order applies only to designs where response format was manipulated within subjects, as a between-subject design would assign only one response format to each subject (i.e., one block of tests with one response format) and therefore, there could be no such condition. For this reason, the testing order (i.e., blocked vs. random) itself would need to be manipulated both within and between subjects, such that some participants experienced both random and blocked testing, and others only one of the two. Again, this was done to investigate whether the retrieval processes involved in both covert and overt retrieval could indeed be considered identical. In a false memory paradigm, Huff et al. (2015) manipulated list order both within and between subjects, and found that free recall performance was better for blocked than for random lists, but only when list order was manipulated within subjects, indicating that there may be carryover effects when testing participants in both blocked and random orders. So if, for instance, we observed a difference in the testing effects created by overt and covert retrieval, depending on whether the participants were subjected to either random or blocked testing – or both – we could conclude that one testing order had an influence on the other. This could happen either by a random test affecting a subsequent blocked test, or vice versa. For this reason, the sequences of testing would need to be fully counterbalanced to avoid order effects. This was the aim of Experiment 4.

## Experiment 1

In Experiment 1, we sought to compare cued recall performance with respect to both a short (∼5 min) and a long (1 week) retention interval, as well as four different learning conditions (*study-only* vs. *covert* vs. *typing* vs. *writing*). The inclusion of a *study-only* condition, which is similar to a control condition, was simply a way of ensuring that the given design did in fact produce a testing effect. In addition to cued recall performance, we also measured response latencies to establish whether they differ by modes of retrieval and/or articulation.

### Method

#### Participants, Design, and Materials

Thirty-two (11 males) participants, with a mean age of 27.19 years (*SD* = 8.95, *range* 19–59), were recruited from various academic disciplines and different universities, institutes and colleges throughout the municipality of Stockholm. For their participation in the study they received either course credit or a movie voucher.

The experiment was designed using E-prime 2.0 professional software (Psychological Software Tools, Pittsburgh, PA, United States) and was run on desktop computers. The stimulus list consisted of 48 word pairs (e.g., flicka - pojke) taken from Swedish Associations Norms (Shaps et al., 1976) that had similar association values (varying from one to three). The association value was computed by Shaps et al. (1976), where participants reported the first word they associated with a certain word they were presented with. An association value *of x* meant that out of 100 participants, *x* individuals reported a specific word associated with a target word. All items in the stimulus list had an association value of two.

#### Procedure

Participants were presented with a written consent form and general description of the experiment was provided. After starting the computer script, their age and gender was entered, and all further instructions were thereafter displayed on the computer screen. The experiment consisted of three phases:

##### Study phase

In the study phase, participants were allowed to study each word pair individually for 6 s, in a random order, and this process was repeated for a total of three times. Between each block of 48 items, a distractor task was given, in which participants would verify as many mathematical expressions as possible in 30 s. This was done by pressing “1” on the keyboard for a correct mathematical expression, and “0” for an incorrect expression.

##### Testing phase

The testing phase contained four separate conditions that were manipulated within subjects. The 48 items were randomly, but evenly, assigned to four conditions, meaning that each condition contained a subset of 12 items which were all displayed or tested in a random order. The four conditions were *covert*, *type, write*, and *study-only.* The study condition contained a fourth opportunity to study each item after the study phase. For the other three conditions, a two-step testing procedure was adopted, with slight variations depending on condition.

First, a cue word was shown to the participants. This is the left word in the word pair, and participants were instructed to try and remember the right (target) word. If they believed they would be able to answer, they would press the ENTER key within 5 s. If this was not done, the script would move on to the next item. If ENTER was, however, pressed within 5 s, participants would either write or type their answer, or do nothing at all, depending on condition. The script ensured that each item would be presented for a total of 12 s, so pressing ENTER after 5 s would leave 7 s to give an answer. Similarly, pressing ENTER after 3 s would leave 9 s to provide the answer, and so on.

In the *covert* condition, pressing ENTER meant that one would have to wait for the remainder of the 12-s period for that particular item. Although time-consuming, this was the only way to ensure that exposure time did not differ between different items and conditions. For this reason, the items in the study condition were also displayed for 12 s.

In the *type* condition, participants were prompted to type their answer on the keyboard after they had pressed ENTER the first time. When finished, they would submit their answer by pressing ENTER again.

In the *write* condition, participants would instead write their answer (i.e., the target word) on a sheet of paper in front of them, and then press ENTER again. Apart from the way the answer was articulated, the procedure was identical to that of the *type* condition.

##### Final recall phase

After having completed the testing phase, participants were given an on-line typing speed test^1^, in which the task was to copy a template text verbatim in 1 min. When 1 min had passed, a score was given that reflected the number of words that the participant had correctly copied. This test was taken three times, and the highest of the three scores was noted.

The typing test served as a short retention interval for the first of the two final cued-recall tests. In these tests, six words from each condition (i.e., half of the items) were selected randomly to be tested at both the short (5 min) and the long (7 days) retention interval. A cue word was shown and participants were given 15 s to type their answer on a keyboard and press ENTER to submit the answer. After 1 week, participants returned to take the final cued recall test, which contained the other half of the items.

### Results

An alpha level of 0.05 was used, and for the analyses of variance (ANOVA) effect sizes are denoted by partial eta squared ($\eta_{p}^{2}$) or Cohen’s *d*.

#### Cued Recall during Learning

Given the design of the experiment, data only allowed for comparison of the cued recall performance during learning, between two of the four learning conditions. This is because no articulation took place for the *study-only* and *covert* conditions. Remember that during learning, the participants pressed the ENTER button when (and if) they had recalled an item, but thereafter, only the *type* and *write* conditions allowed participants to articulate their responses (for the *study-only* condition, no action was required from the participants). However, upon closer inspection of the number of ENTER presses associated with each condition, there appears to be little difference at least in the proportion of affirmative responses across conditions. On average, subjects pressed ENTER equally often for items that belonged to the *covert* (F_3,96_ = 20.35, $\eta_{p}^{2}$ = 0.39, p = 0.001), *type* (M = 10.00;SD = 2.00)and *write (M = 10.03;SD = 2.02)* conditions, that is, roughly 84% of all trials.

There was no significant difference in the cued recall performance between the *type* condition (M = 0.76;SD = 0.20) and the *write* condition (M = 0.74;SD = 0.26) during learning, *t*_31_ < 1. For affirmative responses (i.e., ENTER presses within the specified time frame), recall was generally high for both the *type* (M = 0.92;SD = 0.17) and *write* (M = 0.86;SD = 0.22) conditions. Again, these differences were not significant.

#### Final Cued Recall

A response format × retention interval repeated measures ANOVA on cued recall data showed significant main effects of retention interval, F_1,31_ = 178.05, $\eta_{p}^{2}$ = 0.85, p = 0.001, and response format, F_3,93_ = 5.98, $\eta_{p}^{2}$ = 0.16, p = 0.001, as well as their interaction, F_3,93_ = 8.97, $\eta_{p}^{2}$ = 0.22, p = 0.001. As can be seen in **Table 2**, the conditions *study-only, covert*, and *write* did not differ at the short retention interval, although the *type* condition differed significantly from the covert (t_31_ = 2.58, p = 0.015) and write (t_31_ = 2.92, p = 0.006) conditions, but not the *study*-*only* condition, *t*_31_ < 1. At the long retention interval, the cued recall performance of the conditions *covert*, *type*, and *write* all differed significantly from the *study-only* condition (covert : t_31_ = 4.48, p = 0.001; type : t_31_ = 3.49, p = 0.01; write : t_31_ = 4.56, p = 0.001) but not each other, *ts*_31_ < 1.

**Table 2:** Cued recall performance as a function of the response format and retention interval (with standard deviations in parentheses).

Response format	Retention interval			
	Short		Long	
Study-only	0.80	(0.27)	0.29	(0.22)
Covert	0.77	(0.27)	0.52	(0.32)
Type	0.85	(0.16)	0.47	(0.32)
Write	0.76	(0.26)	0.50	(0.31)

Sidak *post hoc* comparisons revealed that the mean of the *study-only* condition differed significantly from those of all other conditions (*covert: M*_I-J_ = 0.10; *SE* = 0.03, *p* < 0.05; *type: M*_I-J_ = 0.12; *SE* = 0.03, *p* < 0.01; *write: M*_I-J_ = 0.08; *SE* = 0.03, *p* < 0.05). This suggests that the effect was mainly driven by the *study-only* condition relative to the other three conditions.

### Response Latencies during Learning and Final Recall

The *study-only* condition had no measurable response latencies during the learning phase (both cue and target words were shown for 12 s), and was thus excluded from this comparison. For the short and long retention intervals, however, the response latencies of all four conditions are displayed in **Table 3** below. As response latency measurements often yield non-parametric data (as was the case in this experiment), the response latencies are presented in median rather than mean values.

**Table 3:** Median response latencies, in milliseconds, during learning and final recall.

Response format	Retention interval		
	Learning phase	Short	Long
Study-only	-	3361	7070
Covert	2484	3363	6351
Type	2543	3399	6206
Write	2645	3430	6615

Participants had larger response latencies after longer than shorter retention intervals, which is to be expected as a result of forgetting. A Wilcoxon signed-ranks test revealed that at the short retention interval, the response latencies for items that were only studied were higher than those of items that were tested, but only with respect to the write condition, *Z* = 1.81, *p* = 0.07. No significant differences were found at the long retention interval.

### Discussion

The results of Experiment 1 showed a clear testing effect, but its magnitude was not affected by response format in that the covert or overt (i.e., type and write) conditions showed comparable levels of delayed recall, measured in terms of response accuracy and latency. However, these findings do not rule out the possibility of a difference in the relative efficacy of covert vs. overt retrieval. Specifically, a testing effect produced by only one testing session (during learning) may not be sufficiently sensitive for detecting potential effects of response format. This possibility was further investigated in Experiment 2.

## Experiment 2

In Experiment 2, we wanted to ascertain whether the lack of difference in cued recall performance, with respect to the *covert* vs. *type* vs. *write* conditions, would remain even if the magnitude of the testing effect itself was increased. To this end, we included three consecutive testing sessions during initial learning.

### Method

#### Participants, Design, and Materials

Thirty three (10 males) participants, with a mean age of 23.97 years (*SD* = 5.63, *range* 19–43), were recruited from Stockholm University. None of the participants had experience of similar experiments. For their participation, they received either course credit or a movie voucher.

As Experiment 2 was a continuation of Experiment 1, its design and implementation was practically identical to that of Experiment 1, except for certain key differences that will be outlined below.

#### Procedure

The procedural differences between Experiments 1 and 2 were threefold: (i) the exposure time during the study phase was set to 5 s per item (6 s in Experiment 1), (ii) the total exposure time for each item during the testing phase was set to a total of 10 s (12 s in Experiment 1), and (iii) the test phase now consisted of three consecutive testing sessions (one session in Experiment 1). The exposure time shortened to reduce fatigue due to the additional testing sessions.

As the design of Experiment 2 implied that each item would be tested more than once during the testing phase, steps were taken to ensure that the assignment of items to different response formats remained constant across the three testing sessions (although the order of testing for each item was always random). This means that an item, which was tested covertly in the first session, would consequently also be tested covertly in the second and third sessions. Between each testing session, a 30-s distractor task was administered.

### Results

#### Cued Recall during Learning

The number of ENTER presses was entered as the dependent variable into a response format × session order repeated measures ANOVA. There was a significant main effect of session order, F_2,64_ = 11.93, $\eta_{p}^{2}$ = 0.27, p = 0.001, which simply indicates that participants pressed ENTER with increasing frequency across testing sessions. There was no significant main effect of response format, and no response format × session order interaction. As in Experiment 1, there appears to be no differences in the proportion of affirmative responses across conditions, suggesting that subjects responded equally often for all items in the covert, type and write conditions.

There was also no significant difference in the cued recall performance between the *type* condition (M_1st_ = 0.69;SD = 0.20; M_2nd_ = 0.73;SD = 0.21; M_3rd_ = 0.74; SD = 0.22) and the *write* condition (M_1st_ = 0.64;SD = 0.23; M_2nd_ = 0.69;SD = 0.23; M_3rd_ = 0.70;SD = 0.23) during learning. A repeated measures ANOVA with cued recall performance as the dependent variable and response format × session order as the independent variables showed a significant main effect of session order, (F_2,64_ = 10.09, $\eta_{p}^{2}$ = 0.24, p = 0.001), which indicates that participants’ mean cued recall performance increased with each testing session. No other effects were observed.

#### Final Cued Recall

Cued recall performance was entered as the dependent variable into a response format × retention interval A repeated ANOVA on the final cued recall data showed significant main effects of retention interval, (F_1,32_ = 86.80, $\eta_{p}^{2}$ = 0.73, p = 0.001), and response format, (F_3,96_ = 3.73, $\eta_{p}^{2}$ = 0.10, p = 0.01), and their interaction, (F_3,96_ = 20.35, $\eta_{p}^{2}$ = 0.39, p = 0.001).

As evident from **Table 4**, the conditions, *covert*, *type* and *write* did not differ at the short retention interval, although the *study-only* condition differed significantly from the covert (t_32_ = 3.63, p = 0.001) and write (t_32_ = 3.71, p = 0.001) conditions, and only marginally from the *type* condition (t_32_ = 1.90, p = 0.07). At the long retention interval, the conditions *covert*, *type* and *write* all differed significantly from the *study* condition (*covert*: t_32_ = 6.10, p = 0.001; type : t_32_ = 5.40, p = 0.001; write : t_32_ = 4.97, p = 0.001) but not each other (ts_32_ < 1). Again, this suggests that the interaction is driven mainly by differences in the *study-only* condition relative to the other three conditions.

**Table 4:** Mean (*SD*) proportional cued recall performance after the short and the long retention interval as a function of the response format.

Response format	Retention interval			
	Short		Long	
Study-only	0.85	(0.18)	0.32	(0.20)
Covert	0.72	(0.24)	0.61	(0.29)
Type	0.77	(0.22)	0.58	(0.27)
Write	0.72	(0.23)	0.55	(0.30)

#### Response Latencies during Learning and Final Recall

Because Experiment 2 featured three consecutive testing sessions during the learning phase, the mean response latencies decreased across sessions, which indicates that participants became more familiar with the material and thus responded quicker (see **Table 5**). As in Experiment 1, we again observed increased response latencies after 1 week, which simply reflects forgetting. A Wilcoxon signed-ranks test revealed no differences in response latency among the three testing conditions, whereas the study-only condition yielded significantly larger response latencies than all three testing conditions at the long retention interval, *Z*_covert_ = 3.72, *p* = 0.0001; *Z*_type_ = 3.72, *p* = 0.0001; *Z*_write_ = 3.19, *p* = 0.001. At the short retention interval, the study-only condition differed significantly from only the type condition, *Z* = 2.30, *p* = 0.02. Again, this shows that the response latencies are highly similar for all conditions, except for the study-only condition.

**Table 5:** Median response latencies, in milliseconds, during learning and final recall.

Response format	Retention interval				
	Learning phase			Short	Long
	1st	2nd	3rd		
Study-only	-	-	-	3655	6956
Covert	2304	1813	1553	3396	5304
Type	2311	1798	1649	3354	5097
Write	2409	1775	1793	3360	5550

### Discussion

The design and purpose of Experiment 2 were identical to those of Experiment 1, the only difference being the number of testing sessions that produced the testing effect. Consequently, the overall memory performance was greater than in Experiment 1, which is not very surprising. Similarly, we observed lower response latencies than in Experiment 1, again suggesting that participants had better memorized the material over the course of three testing sessions as opposed to only one, in Experiment 1. The results do not suggest any differences in the magnitude of the testing effects produced by the covert and overt response formats. However, we believe it would be premature to simply conclude that there are no differences in the relative efficacy of covert vs. overt retrieval, that is, that the testing effect is entirely driven by retrieval processes (cf., Putnam and Roediger, 2013). The fact that Jönsson et al. (2014) did find an advantage for overt retrieval, and with a moderate effect size (Experiment 1: *d* = 0.25; Experiment 2: *d* = 0.21), coupled with differences in the design of the aforementioned studies, suggests that the relative efficacy of covert vs. overt retrieval may not always reveal itself, if it exists at all. Two key differences that remain to be addressed are the order in which covert and overt testing is performed, namely in blocks or random order, as well as the utilization of within- or between-subjects designs, which will be more closely examined in Experiments 3 and 4.

Moreover, it seems that the two forms of overt testing used in Experiments 1 and 2 (i.e., typing vs. writing) are not contributing differentially to the testing effect, as was hypothesized on the basis of the findings of Mangen et al. (2015). This also means that the TAP hypothesis cannot be confirmed by the findings of these two experiments. A possible explanation for this is that on a practical level, participants may have been so preoccupied by the act of switching between typing their answers on a keyboard and writing them down on paper – from one item to another – that whatever relative efficacy may exist between the two was effectively lost because of the procedure used in these experiments. For these reasons, we used only typing for the overt testing condition in Experiments 3 and 4.

## Experiment 3

The aim of Experiment 3 was to address the possibility that the order in which the tests were administered may have affected the magnitude of the testing effect. As the testing procedure (during learning) in Experiments 1 and 2 was always chosen at random, Experiment 3 included a condition in which items are tested in blocks of either covert or overt tests. As the typing/writing distinction included in Experiments 1 and 2 did not show any significant differences with respect to the testing effect produced, only one overt response format (typing) will be included in Experiment 3.

### Method

#### Participants, Design, and Materials

Forty two (15 males) participants, with a mean age of 24.84 years (*SD* = 6.57, *range* 18–49), were recruited from Stockholm University. For their participation, they received either course credit or a movie voucher.

Experiment 3 was highly similar to Experiments 1 and 2, except for small changes in the design. We included only the covert and overt (typing) conditions, and introduced two ways of ordering these tests, namely in a random or blocked fashion. The list of items was randomly split in two halves, each assigned to either the random or the blocked testing procedure. The covert and overt testing of these items was identical to that of Experiments 1 and 2, with the only difference being the order in which they were tested (i.e., either randomly or in separate blocks of covert and overt testing).

#### Procedure

After having completed three study phases identical to that of Experiments 1 and 2, participants were tested covertly for 24 (i.e., half) of the items, and overtly for the other 24. Each set of 24 items was randomly chosen, and then subdivided into two sets of 12 items, one to be tested in a random order, and the other in blocks of covert and overt testing. This meant that 24 items were tested in blocks of 12 covert and 12 overt items (i.e., 12 consecutive covert followed by 12 consecutive overt tests, or vice versa), and 24 items were tested either covertly or overtly in random order. This 2 × 2 subdivision of items was fully counterbalanced, so that the testing phase would begin with a random testing session equally as often as a blocked testing session, and that equally as many blocked sessions began with covert testing as with overt testing. The blocked tests were alternating, so that a block of covert testing was always followed by a block of overt testing, and vice versa.

### Results

#### Cued Recall during Learning

As in Experiments 1 and 2, there appears to be no difference in the proportion of affirmative responses across conditions during learning. The number of ENTER presses was entered as the dependent variable into a response format × testing order × session order repeated measures ANOVA. There were no significant main or interaction effects, indicating that participants were equally likely to press ENTER for any given item, during the tests in the learning phase, regardless of response format in each respective testing session.

Since covert retrieval, by definition, does not entail the articulation of an answer, there was no way of establishing the proportion correct responses that likely preceded the participants’ ENTER presses in the covert testing conditions. However, there is no reason to suspect that the proportion correct responses, had they been articulated, would be any different from those of the overt condition M_1st_ = 0.91; SD = 0.12; M_2nd_ = 0.91; SD = 0.12; M_3rd_ = 0.92; SD = 0.10.

In order to assess any effects that may have arisen, during learning, from the blocked or random testing conditions, or from the repeated testing sessions, recall performance was entered as the dependent variable into a testing order (block vs. random) × session order (1st, 2nd, or 3rd) repeated measures ANOVA. There were no significant main effects of either testing order or session order, although it should be mentioned that there was a trending main effect of session order, F_2,82_ = 2.45, $\eta_{p}^{2}$ = 0.06, p = 0.09, such that recall performance increased with each consecutive testing session, regardless of testing order.

#### Final Cued Recall

Cued recall performance was entered as the dependent variable into a response format × retention interval × testing order repeated measures ANOVA. There was a significant main effect of retention interval, F_1,41_ = 36.69, $\eta_{p}^{2}$ = 0.47,p = 0.001, which again reflects forgetting over time. The main effect of response format approached statistical significance, F_1,41_ = 3.54, $\eta_{p}^{2}$ = 0.08, p = 0.067. There was no significant main effect of list order. In addition to the main effects, there was also a reliable retention interval × response format interaction, F_1,41_ = 9.34, $\eta_{p}^{2}$ = 0.19, p = 0.004, as well as a response format × testing order interaction, F_1,41_ = 4.20,$\eta_{p}^{2}$ = 0.09, p = 0.047. The three-way interaction was not statistically significant.

**FIGURE 1:**
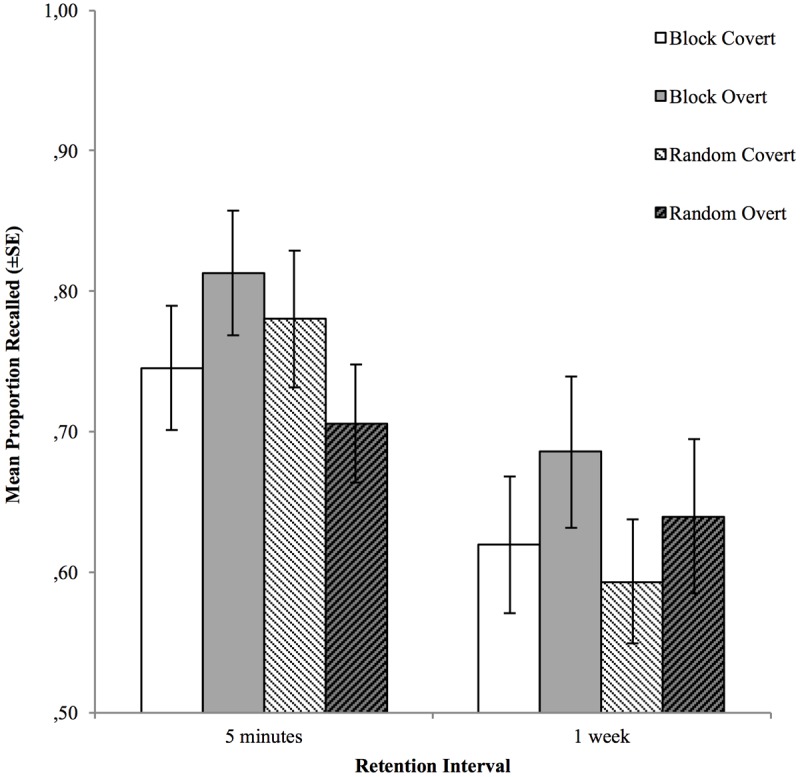
Mean cued recall performance for items tested, covertly and overtly, in blocks and random order, at the two retention intervals. Error bars represent the standard error of the mean.

To follow up on the interaction effects, and since the three-way interaction was not significant, separate response format × testing order ANOVAs were run for the short and the long retention interval. For the short retention interval, both main effects were non-significant, but the response format × testing order interaction was significant, F_1,41_ = 5.03, $\eta_{p}^{2}$ = 0.11, p = 0.03, meaning that the relative efficacy of covert vs. overt retrieval was reversed when testing in random order (see **Figure 1**). At the long retention interval, there was a significant main effect of response format, F_1,41_ = 9.35, $\eta_{p}^{2}$ = 0.19,p = 0.004, which suggests that the relative efficacy of the two response formats is in favor of overt retrieval. Additionally, the memory performance for covert and overt retrieval, across both RIs, were compared separately for blocked and random testing order in paired samples *t*-tests. For blocked tests, overt retrieval led to better overall memory performance, t_41_ = 2.58, p = 0.01, however, for random tests, this difference was not significant, t_41_ < 1.

#### Response Latencies during Learning and Final Recall

Participants had smaller response latencies with each consecutive testing session (see **Table 6**). During both learning and final recall, response latencies were generally larger for items that were tested covertly than overtly tested items. At final recall, larger response latencies were again observed at the long retention interval. A Wilcoxon signed-ranks test revealed that at the short retention interval, covert testing yielded larger response latencies than overt testing, but only for the blocked condition, *Z* = 2.53, *p* = 0.01. At the long retention interval, covert testing also yielded larger response latencies, but only for the random condition, *Z* = 3.01, *p* = 0.003.

**Table 6:** Median response latencies, in milliseconds, during the three testing sessions of the learning phase, and at the two retention intervals.

Response format	Learning phase						Retention interval			
	1st		2nd		3rd		Short		Long	
	Block	Random	Block	Random	Block	Random	Block	Random	Block	Random
Covert	2027	2037	1543	1486	1327	1337	3042	3110	5145	5585
Overt	1860	1901	1392	1515	1249	1323	2855	2933	4814	4718

### Discussion

The aim of Experiment 3 was to elaborate on possible differences in the design of previous studies that may explain or help to reconcile their contrary results. More specifically, we examined whether testing items in blocks or in a random order would have any effect on the testing effect produced. Given the results, it appears that testing order alone cannot account for this relative efficacy. Similarly to the findings of Jönsson et al. (2014), there was an advantage for overt retrieval over covert retrieval, although it was only significant at the long retention interval. Moreover, list order interacted with response format at the short retention interval in such a way that random testing appeared to reverse the advantage for overt vs. covert retrieval, compared to testing in blocks. Across both retention intervals, the advantage for overt retrieval was only present when items were tested in blocks. Thus, it seems that list order may influence how participants perceive and engage in the task of memory testing, however, as the three-way interaction was not significant, it remains difficult to assess the role of list order with respect to the testing effect itself [because it is typically demonstrated by means of a response format × retention interval interaction (c.f., Kornell et al., 2011; Rowland, 2014)].

When shown a cue word, we assume that participants engage in a retrieval process which, if successful, dictates the subsequent responses on the keyboard. If tested in blocks, participants would likely have gotten used to the kind of testing that was currently utilized, and thus take into account the actions following the press of the ENTER button (i.e., nothing, in the case of covert testing, or the articulation of an answer, in the case of overt testing). This knowledge gives the participants the ability to adjust or even cheat in the case of covert testing. That is, if they know they will not be prompted to articulate an answer, they may press the ENTER button with no consequences. If the testing is in random order, however, this possibility is effectively ruled out, as each item has the potential to be tested both covertly and overtly. Therefore, when tested randomly, we should have expected the ENTER button to be pressed more sparingly and only when participants were fully certain of the answer.

To further explore this notion, we compared final memory performance for participants in Experiment 3, this time using the first presented list order as a independent variable. The idea was to establish whether the initial form of testing (i.e., whether it was presented randomly or in blocks) could have an effect on subsequent retrieval or testing efforts. Cued recall performance was thus entered as the dependent variable into a response format (within: covert vs. overt) × retention interval (within: 5 min. vs. 1 week) × testing order (within: block vs. random) × first testing order (between: block vs. random) mixed ANOVA. We only report here the effects of first testing order, as the effects of all within-subjects variables have already been reported earlier. Although there were no significant effects, an interesting response format × first testing order interaction was found, F_1,40_ = 2.94, $\eta_{p}^{2}$ = 0.07, p = 0.094, which – if significant – would have suggested that participants who first experienced randomly presented covert and overt tests tended to perform better on overt than covert tests, whereas participants who first experienced tests presented in blocks tended to perform better on covert than overt tests. But as the effect is non-significant, no conclusions should be drawn from it.

In addition to effects of list order, we also investigated whether the first response format experienced by participants would have any effects in ways similar to those described above. For this reason, only the participants who were first presented with blocked tests were included in the analysis, and divided into those who were first tested covertly, and those who were first tested overtly. The mixed ANOVA showed that there was a retention interval × first test type interaction F_1,17_ = 5.48, $\eta_{p}^{2}$ = 0.24, p = 0.032, such that participants who were first tested covertly appeared to forget less over the course of a week than did participants who were first tested overtly. However, as this comparison is only based on roughly half of the participants, along with the fact that this effect pertains only to the first block of tests administered – and not the tests *per se* – we have chosen not to draw any conclusions from it. It does nonetheless suggest that the way in which participants engage in the testing sessions may differ as a function of what the participants believe the task involves.

The absence of testing order effects suggests that the processes underlying the decision to press the ENTER button are not affected by the prospect of having to articulate an answer (or not). There is, however, another possible explanation for this finding, which resides in the within-subjects design of this experiment. The fact that participants engaged in both random and blocked testing may have led to one form of testing affecting the other, such that participants may choose to err on the side of caution, which would cause them to use the same retrieval strategies and employ the same thresholds for pressing the ENTER button in all testing sessions, regardless of the condition. By directly comparing list order manipulations either within or between subjects, we may not only circumvene the problems mentioned above, but also help explain the somewhat different findings of the two experiments by Jönsson et al. (2014), where both within- and between-subject designs were used. This was the aim of Experiment 4.

## Experiment 4

In Experiment 4, we further explored possible effects of the order of testing, this time for both between- and within-subjects designs. The idea was that any differences, in terms of the testing effect that arises from covert and overt tests that are ordered either randomly or in blocks, may be suppressed by the within-subjects design of Experiment 3. Since every participant was repeatedly tested (covertly and overtly) both in blocks and in random order, it is possible that one mode of testing affected the other. Therefore, we separated the blocked and random-order tests between subjects.

### Participants, Design, and Materials

Sixty-four (13 males) participants, with a mean age of 25.95 years (*SD* = 6.73, *range* 19–54), were recruited from Stockholm University. For their participation, they received either course credit or a movie voucher.

### Procedure

Participants completed the same learning phase as in Experiment 3, but for half of the participants, items were randomly assigned to alternating blocks of covert and overt testing during learning. The other half were tested covertly and overtly on all items such that the response format for each item was randomly selected. This allocation of items to the covert or overt testing format was of course identical in the following two testing sessions of the learning phase.

### Results

#### Cued Recall during Learning

Cued recall performance was entered as the dependent variable into a testing order (*between*: block vs. random) × session order (*within*: 1st, 2nd, or 3rd) mixed ANOVA. There was a significant main effect of session order, F_2,62_ = 32.15, $\eta_{p}^{2}$ = 0.34, p = 0.001 such that recall performance increased with each consecutive testing session, regardless of testing order. There were no significant interaction effects. Sidak *post hoc* comparisons revealed that recall performance was significantly improved for each consecutive testing session (*1st*–*2nd: M*_I-J_ = 0.35; *SE* = 0.01, *p* = 0.001; *2nd–3rd: M*_I-J_ = 0.16; *SE* = 0.01, *p* = 0.01).

As noted before, there are no data available for the memory performance of the covert conditions during learning. The number of ENTER presses were therefore used as a proxy for this measure by entering it as the dependent variable into a testing order (*between*: block vs. random) × session order (*within*: 1st, 2nd, or 3rd) × response format (*within*: covert vs. overt) mixed ANOVA. There was a main effect of session order, F_2,62_ = 34.49, $\eta_{p}^{2}$ = 0.36, p = 0.001, which simply demonstrates that participants tended to press ENTER more often as they learned more of the material across the three testing sessions. There were no other significant main or interaction effects, again suggesting that covert items were likely remembered equally as well as the overt items at the time of testing. Sidak *post hoc* comparisons revealed that the main effect of session order was mainly driven by the difference between the first and the second session (*M*_I-J_ = 0.89; *SE* = 0.13, *p* = 0.001).

#### Final Cued Recall

Cued recall performance was entered as the dependent variable into a response format (*within*: covert vs. overt) × retention interval (*within*: 5 min vs. 1 week) × testing order (*between*: block vs. random) mixed ANOVA. There was a significant main effect of retention interval, F_1,62_ = 45.50, $\eta_{p}^{2}$ = 0.42, p = 0.001, which again reflects forgetting over time. There were no significant main effects of testing order or response format. There was also a significant retention interval × response format interaction, F_1,62_ = 8.50, $\eta_{p}^{2}$ = 0.12, p = 0.005. The three-way interaction was not statistically significant.

**FIGURE 2:**
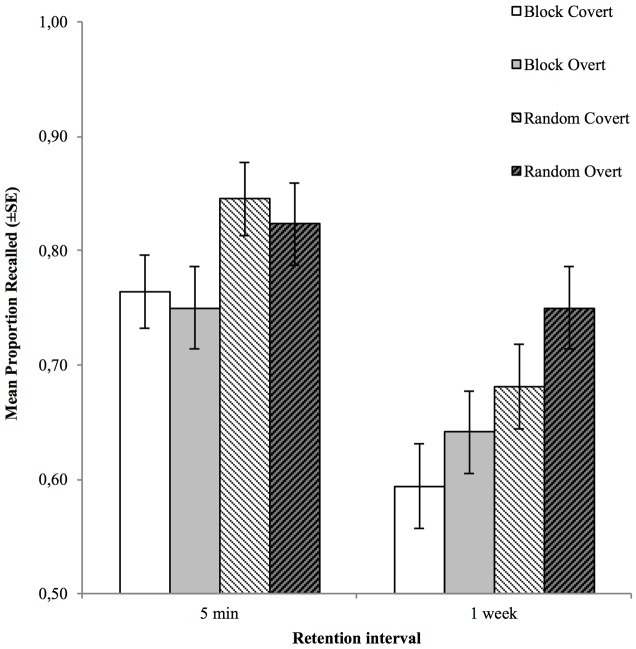
Mean cued recall performance for items tested, covertly and overtly, in blocks and random order, at the two retention intervals. Error bars represent the standard error of the mean.

To follow up on the interaction effects, and since the three-way interaction was not significant, separate response format × testing order mixed ANOVAs were run for the short and the long retention interval. For the short retention interval, there were no significant effects whatsoever, but for the long retention interval, there was a significant main effect of response format, F_1,62_ = 7.95, $\eta_{p}^{2}$ = 0.11, p = 0.006, suggesting a relative efficacy in favor of overt vs. covert retrieval (see **Figure 2**).

#### Response Latencies during Learning and Final Recall

The pattern of response latencies was highly similar to that of Experiment 3 (see **Table 7**), with the same decrease with each consecutive testing session, along with larger response latencies after the long retention interval compared to the short. A Wilcoxon signed-ranks test revealed that covert testing yielded larger response latencies than overt testing for both the blocked and random conditions, *Z*_block_ = 2.59, *p* = 0.01; *Z*_random_ = 2.42, *p* = 0.02, but only at the long retention interval.

**Table 7:** Median response latencies, in milliseconds, during the three testing sessions of the learning phase, and at the two retention intervals.

Response format	Learning phase						Retention interval			
	1st		2nd		3rd		Short		Long	
	Block	Random	Block	Random	Block	Random	Block	Random	Block	Random
Covert	1986	1817	1640	1417	1388	1337	3027	3227	5673	5401
Overt	1872	1890	1477	1553	1300	1306	2959	2987	4203	4865

### Discussion

Experiment 4 was identical to Experiment 3, apart from the manipulation of testing order between subjects instead of within. Results show that similarly to Experiment 3, this experiment did not demonstrate a main effect of response format (although this effect approached statistical significance in Experiment 3). Both Experiments 3 and 4 do confirm, however, that there appears to be an advantage for overt vs. covert retrieval, with respect to the testing effect produced, at the long retention interval, as confirmed by the interaction effects. This finding is in line with the results reported by Jönsson et al. (2014). However, the list order manipulation appears to have had no effect on the testing effects created by covert and overt retrieval. In **Table 1**, we have compiled all the relevant testing-effect studies in which covert and overt retrieval have been compared directly. The main results of the four experiments of this paper have also been included in this table.

## General Discussion

As we have seen in these four experiments, the evidence for a relative efficacy of covert vs. overt retrieval remains equivocal and marginal. Although Experiments 1 and 2 demonstrated a testing effect, it did not differ in magnitude depending on the response format. This is in line with the findings of Putnam and Roediger (2013), but not with those of Jönsson et al. (2014), which are similar to what was found in Experiments 3 and 4. As noted before, the disparity in these findings may be explained in a number of ways, and the four experiments of this paper have focused mainly on several differences in the design of previous studies (see **Table 1**). More specifically, the type of articulation has been manipulated (in Experiments 1 and 2), as well as the order of testing (random vs. blocked; Experiment 3) and whether this was manipulated within or between subjects (Experiment 4).

The findings of this study clarified that the testing order manipulation seems to have had little to do with the magnitude of the testing effect created by covert and overt testing. This also appears to be the case for both within- and between-subjects manipulation of the testing order (although in Experiment 3, there was a significant response format × testing order interaction at the long retention interval; see **Figure 1**), which is in line with the findings of Rowland et al. (2014). In terms of explaining the contradictory findings within this field, there are now fewer proverbial stones left unturned because of these results. A possible way forward might be a more thorough examination of the retention intervals used in testing effect studies, and the importance of the chosen length of this interval. It can reasonably be assumed that whatever effects have been observed after 1 week, as in the four experiments of this study, are not necessarily the same, in neither magnitude nor direction, as those that may appear after a retention interval of 2 days, for instance.

Similarly, the exposure time during testing may play a more important role than expected, especially when considering that some studies have equated their exposure times across all conditions (e.g., Smith et al., 2013), whereas others allow for additional time to be spent with a particular item, as a result of articulating it (e.g., Jönsson et al., 2014). Since articulation is typically preceded by retrieval, overt forms of testing should provide more exposure to an item than covert retrieval if no action is taken to equate the exposure time, and this in turn may explain why overt retrieval should produce stronger testing effects than covert retrieval.

Another way of minimizing the effects of differences in exposure is by providing feedback during testing (see Rowland and DeLosh, 2015, for a discussion of this). In the current study, however, exposure times were always equated, and no feedback was provided. This effectively makes the covert and overt conditions highly similar to each other, procedurally speaking (this is especially true of the random testing order condition, as participants do not know whether they will be asked to articulate the information after having retrieved it). So, any differences between covert and overt retrieval, with respect to the testing effect, cannot be due to differences in exposure.

A recent and promising line of evidence comes from Tauber et al. (2017), who compared covert and overt retrieval for more complex materials than paired associates, namely key-term definitions. Their participants learned key-terms definitions and then either restudied or practiced retrieving them covertly or overtly. At final recall (48 h later), the overt retrieval group remembered significantly more than the study-only and covert retrieval groups. In a second experiment, the covert retrieval instructions were altered so that participants were specifically instructed to “silently retrieve the entire definition for each term,” and with this enhanced form of covert retrieval, the differences between covert and overt retrieval were no longer significant.

Tauber et al. (2017) argue that the benefit of overt retrieval, relative to covert retrieval, is the result of exhaustive or elaborated retrieval, that is, an attempt to fully bring to mind and articulate (internally or overtly) some information, rather than making a familiarity assessment of it. Therefore, in the second experiment, the explicit instructions to silently retrieve the answer discouraged reliance on familiarity assessments, and instead elicited retrieval (albeit without overt articulation) of the sought-after information. Again, this shows that in the context of cued-recall testing, the act of retrieval appears to be the main driver behind the testing effect. It should be pointed out, though, that the nature of the material itself, and its level of complexity, appears to be of importance here. As evident from Experiments 3 and 4, the (covert) random list order conditions should have produced similar retrieval attempts as those in the enhanced covert retrieval conditions in Experiment 2 of the study by Tauber et al. (2017), and yet, we observed no effects of list order. This suggests that there may be a relative efficacy of overt vs. covert retrieval for some learning materials and not for others, and that this difference depends on the degree of elaborated retrieval that is evoked by the material.

### Response Latencies as Indicators of Retrieval Effort

Earlier, we argued that response latency does not directly measure the effort made by a participant to retrieve some information, or the effectiveness of that effort for that matter, the logic being that two processes can be equal in duration and be vastly different in nature. However, looking at the response latencies in these four experiments, we see that the differences found between response format and list order conditions closely mirror the effects found in terms of memory performance.

In Experiments 1 and 2, neither of the testing conditions differed from each other in terms of memory performance, whereas the study-only condition differed from all three. Likewise, the response latencies did not differ for the three testing conditions, whereas the study-only condition differed significantly from all three.

In Experiment 3, the relative efficacy of overt vs. covert was established by a response format × retention interval interaction, and similarly, the response latencies were larger for covert retrieval than for overt retrieval (albeit for different list orders, depending on retention interval). In Experiment 4, the same relative efficacy was indicated by a main effect of response format at the long retention interval, and again this was reflected by the larger response latencies for covert retrieval at the long retention interval.

Taken together, these findings suggest that retrieval latencies may indeed reflect the degree of effort that participants put into retrieving information from memory. They also corroborate the ideas put forth by Jönsson et al. (2014), namely that because the response latencies do not differ significantly, the retrieval processes in covert and overt testing are indeed similar and comparable to one another. By extension, this also means that whatever differences in memory performance that may have arisen between covert and overt testing must be caused by something other than the retrieval processes that the two response formats entail.

## Conclusion

Given the previous findings within this field (e.g., Putnam and Roediger, 2013; Smith et al., 2013; Jönsson et al., 2014), along with the findings of the four experiments of this paper, we conclude that the relative efficacy of covert vs. overt retrieval is not only elusive, in the sense that the effect appears to come and go, but also weak in terms of effect size. As evident from **Table 1**, the average effect size of this comparison, as reported in 13 experiments, is *d* = 0.07 (or *d* = 0.06 if the four experiments of this paper are omitted). The small effect sizes, paired with the inconsistent findings of previous studies, suggest that covert and overt retrieval produce testing effects of comparable magnitudes, especially when the two learning conditions are highly similar to each other. When and if a significant difference is found, it is likely the result of specific design aspects of a particular experiment, rather than an actual difference in efficacy of covert vs. overt retrieval – and even then, the small effect size discourages conclusions that are too far-reaching. In sum, an important conclusion that can be drawn from the present study is that the testing effect is primarily the result of retrieval processes, and that articulation has fairly little to add to, or beyond, what is already produced by retrieval itself.

Naturally, we should point out that these conclusions pertain to the typical design of testing effect experiments, and perhaps not to all real-world settings, where effects of articulation, handwriting, and in more general terms, embodied cognition (e.g., Wilson, 2002, all may have beneficial effects on different aspects of memory. To students who wish to engage in optimal learning behavior, we would simply suggest any form of learning that involves testing because it elicits retrieval. If the effect sizes associated with the relative efficacy of covert vs. overt retrieval were larger, they would have practical implications for the study advice that should be given to students, but as that is not the case, we can only proclaim the importance of retrieval-based learning. Surely articulation may be associated with memorial benefits (e.g., Mangen et al., 2015), but apparently not from point of view of the testing effect itself.

## Ethics Statement

This study was carried out in accordance with the recommendations of the American Psychological Association’s Ethical principles of Psychologists and Code of Conduct. All subjects gave written informed consent in accordance with the Declaration of Helsinki. Given the non-intrusive nature of the methods employed by this study, we, the authors, believe there are no ethical concerns that would need to be reviewed and considered by an ethics committee. The Regional Ethic Review Board, Stockholm (www.epn.se), concluded that the project involved no ethical concerns needed to be further reviewed.

## Author Contributions

The design of the experiments of this paper were developed by MS and FJ. Data collection and analysis was carried out by MS. Drafting the article was done by MS. Critical revision of the article was made by TM and FJ, iteratively with MS. Final approval of the version to be submitted for publication was made unanimously by MS, TM and FJ.

## Conflict of Interest Statement

The authors declare that the research was conducted in the absence of any commercial or financial relationships that could be construed as a potential conflict of interest.

## Footnotes

1 http://10fastfingers.com/typing-test/swedish/
